# Comparative Germination Ecology of Two Endemic *Rhaponticum* Species (*Asteraceae*) in Different Climatic Zones of the Ligurian and Maritime Alps (Piedmont, Italy)

**DOI:** 10.3390/plants9060708

**Published:** 2020-06-02

**Authors:** Valentina Carasso, Marco Mucciarelli, Francesco Dovana, Jonas V Müller

**Affiliations:** 1 Centro Regionale Biodiversità Vegetale, Ente di gestione delle Aree Protette delle Alpi Marittime, Via S. Anna, 34, 12013 Chiusa di Pesio, Italy; 2 Department of Life Sciences and Systems Biology, Viale P.A. Mattioli, 25, Università di Torino, 10125 Torino, Italy; marco.mucciarelli@unito.it (M.M.); francescodovana@libero.it (F.D.); 3 Royal Botanic Gardens Kew, Millennium Seed Bank, Conservation Science, Wakehurst Place, Ardingly, West Sussex RH17 6TN, UK; j.mueller@kew.org

**Keywords:** cold stratification, comparative germination ecology, maritime and ligurian alps, *Rhaponticum*, seed dormancy and germination, soil seed bank, thermoclimatic belts

## Abstract

Comparative studies of seed germination of closely related taxa can help increase our understanding of the ecological limitations of cold-adapted plants and forecast how they might respond to global warming. No studies exist on the relationship between thermoclimatic belts that classify mountain life zones according to bioclimatic criteria and the germination strategy of alpine plants. The aim of this study was to assess this relationship using two closely related species growing in different thermotypes and to test whether their germination responses were related to the climate at natural sites. Fresh *Rhaponticum bicknellii* and *R. scariosum* seeds were cold stratified for 0, 30, 60 and 90 days and tested for germination at 10, 15 and 20 °C. At the same time, seed burial experiments were run in the field and in the plant nursery. A GLM analysis showed that the length of cold stratification affected significantly only the germination of *R. bicknellii* seeds, while increasing temperatures prompted germination in both species. We found that *R. bicknellii* adopts a drought-avoiding germination strategy, while *R. scariosum* germination is favoured by warm temperatures. Our findings support the general view that alpine plants do not share common germination requirements and that any conclusions should be interpreted from a biogeographical and bioclimatic perspective. Therefore, seed germination and seedling establishment of endemic alpine species can also be predicted by looking at the bioclimate of the species’ range.

## 1. Introduction

Seed dormancy plays a pivotal role in timing germination and seedling emergence and establishment [1,2], the most important phases in the life cycle of every plant species. Like other life stages, seed dormancy and germination are sensitive to variations in the environmental conditions [3,4,5]. Depending on variation in flowering time, seed mass, parental investment and maternal effects caused by genetic, epigenetic and/or environmental factors, the mechanisms that regulate seed dormancy release can vary dramatically, even within the same species [6,7]. 

As pointed out by [8], germination depends to a great extent on natural selection pressure, thereby it is associated with the ecological niche and the range of the plant species. Numerous studies have documented that variations in dormancy and germination traits among and within species are associated with the habitat (reviewed in [9]). For example, for some species, germination is impaired by the absence of a particular environmental cue (such as light, fire, low or high temperatures), and the permanent absence of these signals may preclude the establishment of the species in a particular location [8].

It is well known that many alpine plants increase their final germination after a cold period (cold stratification), which is necessary to break the physiological component of dormancy (physiological dormancy) [9]. In fact, dormancy is the result of inhibiting physiological mechanisms that act within the embryo until their complete removal. In nature, embryo inhibition is gradually suppressed after exposure to winter temperatures, and the dormancy status is removed at a rate that depends on the intensity of inhibition [10,11,12,13]. Most alpine species germinate immediately after the snowmelt, while their germination in autumn is normally precluded by the physiological dormancy and the combination of low temperatures and short day lengths [5,14,15].

However, the role and efficacy of the cold stratification for seed germination of alpine species is not always obvious and related to the mean duration of the snow cover in a given habitat. Based on detailed, albeit contrasting studies, it was shown that alpine species are less dormant and require higher germination temperatures than subalpine species [9]. This dependence on relatively high temperatures was interpreted as a reproductive strategy that is used to postpone germination and seedling emergence until late spring or early summer when climatic conditions are more favourable and the seedling is less likely to be exposed to freezing temperatures [16,17]. This finding is documented for many alpine and tundra species that show surprisingly high germination temperature optima. However, rapid germination at relatively high temperatures was also documented for some species in the Mediterranean mountains [18]. Thus, the extent to which germination strategies adopted by different taxa and environmental conditions (associated with their habitats) are correlated is still not fully understood. Yet another germination strategy, characterised by seeds germinating at cold temperatures, was proposed for certain mountain species growing at lower elevations [19]. In fact, seed sensing of winter duration could be crucial for a plant in warmer climates as it allows seed germination to happen immediately after snowmelt when the soil moisture is high and when it is more likely that the seedling can establish itself successfully [19]. Such a strategy could be advantageous for those plants that experience severe drought conditions at the onset of summer, such as plants in many dry parts of the western Alps. 

In brief, many studies suggest that alpine and subalpine species lack a common germination strategy [20] and that their germination requirements can vary greatly over space and time [6,21,22]. It is difficult to associate precise germination traits to specific alpine environments/habitats as this depends also on the number of microhabitats [14,20]. In this regard, small scale variations in temperature and precipitation are just as important as environmental signals.

**Figure 1:**
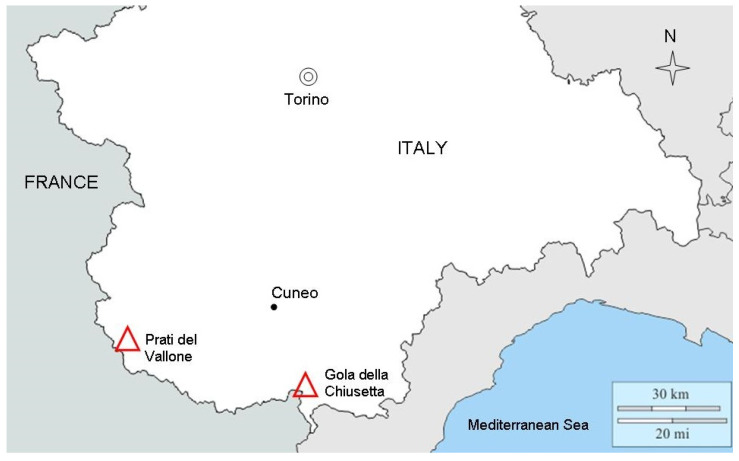
The location of the two study areas in the south-western Alps.

In this paper, we investigated the germination responses of *Rhaponticum scariosum* (Asteraceae), which is endemic to the south-western Alps, and of *Rhaponticum bicknellii,* which is narrow endemic to the Ligurian and Maritime Alps (Figure 1) [23,24]. 

These two species are closely related (Supplementary file S1) but belong to two different thermotypes [25,26,27,28,29,30]. The range of *R. scariosum* falls within the orotemperate belt between 1700 and 2500 m a.s.l. [23,31,32] with a relatively low annual temperature threshold (*Tp* = 381–800 °C, whereby *Tp* is the yearly positive temperature, calculated as the sum of the monthly average temperatures above 0 °C, [24,33]). The range of *R. bicknellii* corresponds to the supratemperate (Submediterranean) bioclimatic belt between 800 and 1700 m a.s.l., with a relatively higher temperature threshold (*Tp* = 801–1400 °C). 

To the best of our knowledge, no previous studies exist which analyse the relationship between thermoclimatic belts (thermotypes) and the germination strategy of rare alpine species. Therefore, the rarity, vulnerability and threat status of the two *Rhaponticum* species makes them ideal candidates to investigate this relationship and, possibly, to forecast the impact of global warming on seed germination. 

Our previous work on *Rhaponticum* endemics showed that the final seed germination of these species increases after a cold stratification (V. Carasso, unpublished data). This finding is in line with the general pattern for species with physiological dormancy.

Our overall hypothesis was that the two *Rhaponticum* species would show different adaptations, in terms of temperature requirements for dormancy break, according to the recorded air temperatures at their growing site. In particular, we expected that (i) both *R. scariosum* and *R. bicknellii* would show physiological dormancy and, specifically, that the orotemperate *R. scariosum* would exhibit (ii) a higher dependence of germination percentages and rates on a period of cold stratification and (iii) a requirement for higher germination temperatures compared to the seeds of the supratemperate *R. bicknellii*. 

In order to test the previous hypotheses, the following studies were done: (1) recording the microclimatic conditions at the growing sites and comparing them with local temperature and precipitation data; (2) converting temperature data into different climatic parameters representative of the local conditions; (3) setting up germination experiments under controlled conditions in the lab; (4) carrying out seed burial experiments at the study sites and in the plant nursery.

## 2. Results

### 2.1. Study 1—Microclimatic Conditions at the Growing Sites and in the Nursery

**Figure 2:**
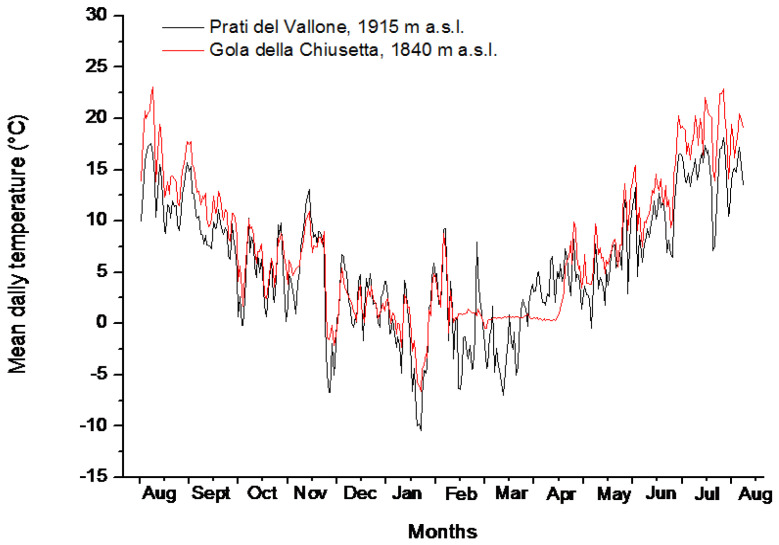
Mean daily temperatures (°C) on the soil surface in Prati del Vallone (PDV, 1915 m a.s.l., dark line) and in Gola della Chiusetta (Gola della Chiusetta—GDC, 1840 m a.s.l., red line) between August 2015 and August 2016.

During the 2015–2016 winter, snow cover only occurred at Gola della Chiusetta (GDC), between the 9th and the 13th weeks of the year (WOY). During this period, the temperature on the soil surface remained constantly close to 0 °C (between + 0.37 °C and + 0.67 °C), with relative humidity values ranging between 6.77% and 9.68% (Supplementary file S2; Figure 2). At Prati del Vallone (PDV), the snow cover was absent during the 2015–2016 winter, and, therefore, the number of weeks with mean temperatures below 0 °C was higher than at GDC (Supplementary file S2; Figure 2). 

However, at GDC, the time period with weekly mean temperatures on the soil surface ≤ + 2 °C lasted for 16 weeks (between the 52th and the 14th WOY), while at PDV, this period only lasted for 12 weeks (between the 53th and the 11th WOY), except in WOY 4 when temperatures at both sites were > 4 °C. The mean weekly temperatures on the soil surface during these periods were + 0.96 °C at GDC and + 0.33 °C at PDV (Supplementary file S2). The mean weekly soil temperatures and Relative Humidity values for the summer of 2015 were + 13.5 °C and 75.68% at GDC and + 14.3 °C and 59.61% at PDV (Supplementary file S2) during the time of seed collecting. 

During the period October 2018–June 2019 at the regional nursery “Gambarello” the soil temperature reached values under 5 °C in the interval between the 47th and the 6 th WOY, except for WOY 49 (5.20 °C). Temperatures >10 °C were recorded between 41st and 43rd WOY and between 15th and 23rd WOY (Supplementary file S3).

### 2.2. Study 2—Conversion of Temperature Data into Different Climatic Parameters

The annual *Tp* values were 921.0 (tenths of degrees) at GDC and 667.5 at PDV, thus falling into the ranges for the (upper) supratemperate (*Tp* = 801–1400) and the orotemperate (*Tp* = 381–800) thermoclimates respectively as defined by [24] (Supplementary file S4). Since *Tp* is the sum of any month of the year with a mean temperature >0 °C, its values are not affected by any differences in soil temperatures which are due to the presence or absence of snow cover, and therefore it allowed us to directly compare the two growing areas. The mean monthly temperatures (°C) were constantly higher at GDC than at PDV during the observation period 2015–2016, that is for the whole growing season defined as those months with an average temperature of 5 °C (Table S4 and Figure S1 in Supplementary file S4). The yearly GDDs were 2978.4 and 2579.7 °C at GDC and PDV, respectively (Table S4 in Supplementary file S4). A comparison of the monthly GDDs in the two study areas showed that the monthly GDDs during the August–October 2015 and May–July 2016 periods were much higher at GDC than at PDV (Table S4 and Figure S2 in Supplementary file S4). This means that a positive difference of 506.9 GDDs characterised GDC by the end of the growing season and there was 1.2–1.3× more heat energy available for growing at GDC than at PDV during the spring-summer transition (May–July). However, consistent with the greater number of weekly average temperatures ≤ + 2 °C during winter and until March at GDC, the monthly GDDs were similar for the two study areas or even slightly higher at PDV (Table S4 in Supplementary file S4).

According to the data from the two meteorological stations, the monthly precipitations in April, May and June were much higher at PDV than at GDC, while GDC showed a higher total precipitation in July, August and October (Table S5 and Figure S3 Supplementary file S4). However, the number of rainy days per month was consistently lower at GDC than at PDV over the whole growth period (Table S5 and Figure S3 in Supplementary file S4). The differences in the distribution of precipitation between the two sites were also consistent with the total number of rainy days registered at the two sites in the 2012–2017 period (Table S5 and Figure S4 in Supplementary file S4). 

### 2.3. Study 3—Germination Experiments under Controlled Conditions in the Lab

Statistically significant differences (*p* < 0.001) were observed between the final germination percentages of the *Rhaponticum* seeds at different incubation temperatures. In addition, the effect of the interaction between *R. bicknellii* and the length of cold stratification was statistically significant (*p* < 0.01) (Table 1 and Table S6a in Supplementary file S5).

No statistically significant differences in the Mean Time to germinate were found between the two species, the incubation temperature or the interaction of these two factors (Table S6b in Supplementary file S5).

Using a GLM (with final germination percentages as the response variable and the length of cold stratification and the incubation temperature as the explanatory variables), the incubation temperature positively affected germination in both species. This effect was statistically significant (*p* < 0.05) for *R. scariosum* and statistically highly significant (*p* < 0.001) for *R. bicknellii* (Table 2 and Table 3 and Table S6c–d in Supplementary file S5). 

Moreover, the length of the cold stratification resulted in a statistically highly significant (*p* < 0.001) effect only for *R. bicknellii* (Table 3 and Table S6d in Supplementary file S5).

**Figure 3:**
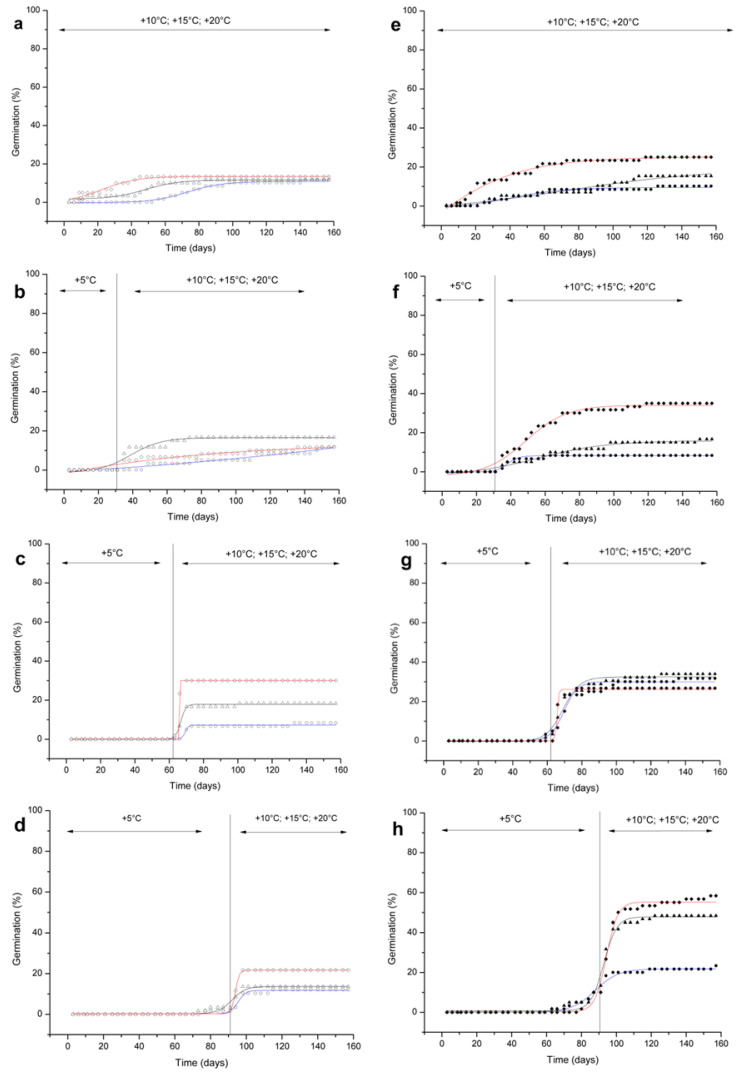
Cumulative seed germination percentages of *R. scariosum* (**a**–**d**; open symbols) and *R. bicknellii* (**e**–**h**; solid symbols). The length of the cold stratification (0, 30, 60, 90 days) and the incubation temperatures (open/solid circles 10 °C, open/solid triangles 15 °C and open/solid diamonds 20 °C) are shown for both species. Sigmoidal curves were fitted with a Boltzmann function.

In both species, without any cold stratification, the first seeds germinated within 30 days of the start of the experiments (Figure 3a,e). 

Final germination percentages were ≤ 15% for all treatments, except for *R. bicknellii*, which reached a final germination of 25% at 20 °C (Figure 3e).

Increasing the length of cold stratification resulted in a prompt germination at 15 °C and 20 °C (Figure 3b–h). In particular, *R. bicknellii* seeds culminated in the highest germination percentage at 20 °C after 90 days of cold stratification (58%) (Figure 3h; Figure S5c in Supplementary file S6), while for *R. scariosum*, the highest germination percentage (30%) was reached after 60 days of cold stratification at 20 °C (Figure 3c, and Figure S5c in Supplementary file S6).

When the seeds were moved to warmer temperatures after 30 days of cold stratification, they started to germinate at once (Figure 3b,f). Very few seeds were already germinating after 60 days (Figure 3c,g) and 90 days of cold stratification (*R. scariosum* 1.69%–1.72%, *R. bicknellii* 1.67%–3.33%) (Figure 3d,h). The germination process was slow at 10 °C. However, the final germination percentages were higher in *R. bicknellii* (≤31.67%) than in *R. scariosum* (≤12.07%) (Figure 3c–h). The final germination percentages increased proportionally at higher temperatures, with the exception of *R. scariosum* after 30 days and *R. bicknellii* after 60 days of cold stratification.

### 2.4. Study 4—Seed Burial Experiments in the Field and in the Plant Nursery

**Figure 4:**
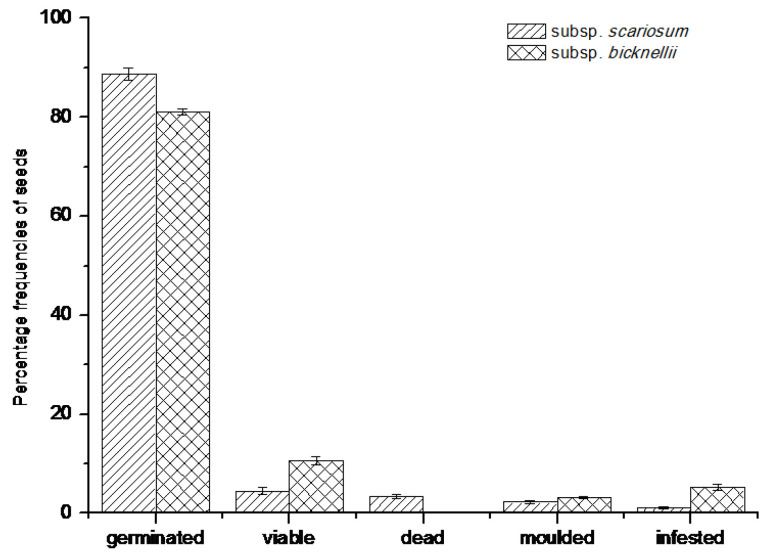
Percentages frequencies of the seed types analysed by means of a cut-test and a Tetrazolium (TZ) test (viable seeds) on seeds retrieved after one year of burial. Data refer to the mean values of five replicates of 20 seeds each ± SE.

For both species, most seeds had germinated in the field by the month of June (Supplementary file S7). Although no intact plantlets were detected inside the nylon mesh sachets, the presence of healthy and firm rootlets and empty seed coats were clear signs of germinated seeds. The final germination percentages (in % ± S.E.) were, 88.76 ± 1.24 for *R. scariosum* and 81.05 ± 0.60 for *R. bicknellii*. For *R. scariosum*, 4.49 ± 0.80 of the seeds were still intact and viable, while for seeds of *R. bicknellii* this percentage was 10.53 ± 0.84 (Figure 4). 

**Figure 5:**
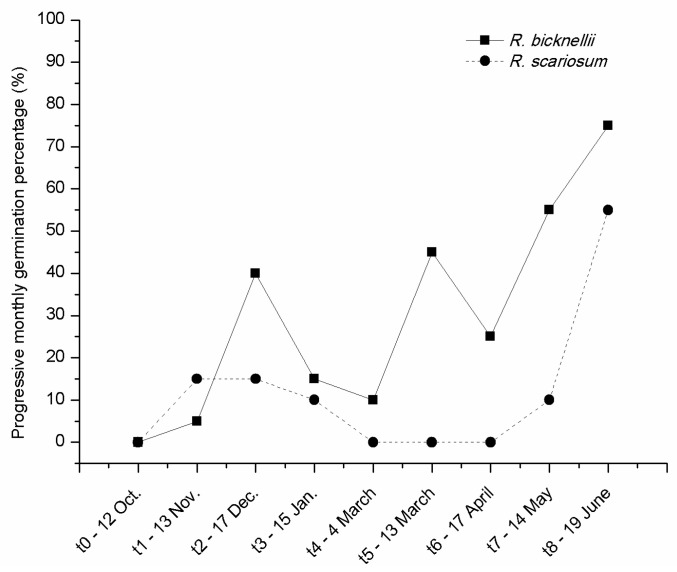
Progressive monthly germination percentages at the nursery of *R. scariosum* (close circle) and *R. bicknellii* (close squares) at different intervals from sowing (t0).

Looking at the curve of progressive monthly germinations of the seeds buried in soil at the plant nursery (Figure 5) one month after sowing, seeds of both species were already germinating (t1-13 Nov.). 

While in *R. bicknellii* two picks of germination are visible, corresponding to the months of December (40%) and March (45%) and germination never stopped, the seeds of *R. scariosum* stopped germinating for two months (March–April, 0%) and reached only in June a value higher than in winter. The maximum final germination percentage was recorded for both species in June (75% for *R. bicknellii* and 55% for *R. scariosum*). 

At the end of each month the recovered seeds were analysed. For *R. bicknellii* the highest percentages of empty seeds were reached in June (75%), May (55%) and March (45%) while the major number of full seeds (except the control) was noticed in November (95%), at the beginning of March (85%) and in February (90%). 

In *R. scariosum* the major percentage of empty seeds was achieved in April, May and June (75%, 60%, and 65%, respectively) while the highest value of full seeds (100%) was recorded in March.

## 3. Discussion

### 3.1. Germination Experiments under Controlled Conditions in the Lab

#### 3.1.1. Seed Responses to Cold Stratification

The results of our laboratory experiments showed significant effects of temperature on seed germination. Within 10 days from the start of the experiments at 20 °C, seeds of *Rhaponticum* spp. were able to germinate even in the absence of a cold stratification, albeit with relatively low final percentages. This finding suggests that only a few seeds were not dormant. 

In a few species from the Chilean Andes, Cavieres and Sierra-Almeida [19] also found germination events without cold stratification, with either high or very low (<10%) final percentages, but at the same time other species that germinated only after a cold and wet period [18]. Several other studies documented a general positive effect of a cold period or stratification [16,18,34,35,36,37,38,39,40,41,42,43]. In our study, the two *Rhaponticum* species showed different and opposite behaviours, and only in *R. bicknellii* the duration of the cold pretreatment showed high statistically significant effects. In fact, in this species, longer cold stratifications were associated with increasing final germination percentages, while the seeds of *R. scariosum* did not respond even to a 90-d cold treatment. This result ruled out our hypothesis of a positive relationship between the duration of cold stratification for maximum germination of this species.

#### 3.1.2. Physiological Dormancy

Our results did not statistically support the interaction between cold stratification and incubation temperatures for either species. These two factors seem to be independent phenomena, at least for *R. bicknellii*, which showed a highly significant response to cold stratification, with possible additive effects on germination. Fresh *R. bicknellii* seeds were able to germinate within 10 days, but only at 20 °C, albeit with very low percentages. Extending the length of cold stratification from 30 to 60 days also induced germination in those seeds incubated at 15 and 10 °C, and a 90-d cold stratification dramatically improved germination at 15 °C and 20 °C but not at 10 °C. Overall, these results point towards the presence of a physiological dormancy in *R. bicknellii* seeds. Moreover, the observed increasing dormancy release as the cold period proceeds could correspond to a situation of conditional or relative dormancy. Relative dormancy is a common phenomenon in seeds with a nondeep physiological dormancy ([13,38] and references therein) and it is documented for other alpine species [18,42]. Its extent is not uniform and not easy to determine in nature, when the differences in the germination ability between nondormant and relative dormant seeds are minimal, and thus most of the seeds germinate [38].

Increasing the length of the cold stratification from 30 to 60 days induced a germination response in *R. scariosum* seeds, which produced final percentages that were proportionally higher for increasing incubation temperatures, despite weak statistical support. This finding partially supports our third hypothesis concerning the response of this species to short durations of cold stratification and warm temperatures for germination. *R. scariosum* seeds need relatively mild temperatures to germinate, in order to avoid to germinate in early spring (or autumn) when a high frost probability would otherwise result in a very low number of established seedlings [16,19,43]. Assuming that it is common of many Compositae to present low percentage of germinated seeds, the reason for the low germination responses of *R. scariosum* in general and in particular after the longest cold treatment however remains unclear. One explanation could be the presence of a deep level of physiological dormancy that we were not able to detect in our study. Mechanisms that regulate dormancy breaking in *R. scariosum* may require the exposure of seeds to a warm stratification, as demonstrated for some alpine species in exposed habitats [41]. 

#### 3.1.3. *R. scariosum* and *R. bicknellii* do not Share Common Germination Strategies

In 1997, a new hypothesis was developed [37] suggesting that the timing of seedling emergence in mountain environments is determined by the ambient temperature and by the amount of rain in spring rather than by the temperature during and the length of the winter. According to this hypothesis, at least two different dormancy syndromes for an appropriate timing of spring germination can be envisaged: (a) species that experience severe drought in late spring and summer react to a long period of cold stratification in order to germinate at low temperatures soon after the snow melt [17,18,43] and, (b) other species that grow at sites with evenly distributed rainfall during summer only require a short period of cold stratification, coupled with an inability to germinate at low temperatures ([43] and references therein), which indeed delays seed germination until the spring or until early summer when temperatures are higher [20].

These observations might explain the observed opposite behaviour of our two species. In fact, higher positive temperatures (*Ti*) and greater accumulated monthly growing degree-days (DGGs) were measured at GDC compared to PDV during the May–October period. This was coupled with significantly fewer rainy days. These circumstances would expose *R. bicknellii* seedlings to the risk of drought in late spring-summer. We therefore have reason to believe that germination in *R. bicknellii* is conditioned by the duration of the cold period during winter, with seedling germination and establishment taking place as soon as the snow melts. In 2016, this happened between the end of April and the beginning of May. In contrast, the seed germination in *R. scariosum,* which experienced a much lower number of GDDs and more evenly distributed precipitation in the late spring-early summer period, was found to be unrelated to the length of the cold period. Seed germination takes place as the temperature begins to rise at the onset of summer (Figure 5) and seedling establishing is favoured by more profuse and diffuse precipitations (on average in May–June). Moreover, unlike *R. bicknellii*, the seeds of *R. scariosum* were unable to germinate at low temperatures.

These considerations are further strengthened by the results obtained by the seed burial experiments. 

### 3.2. Seed Burial Experiments in the Field and in the Plant Nursery

#### 3.2.1. *R. scariosum* and *R. bicknellii* Form Transient Soil Seed Banks

It is well known that seeds of several alpine plants survive in the soil in their natural habitats for many years [42]. However, whether or not a soil seed bank is formed depends on both endogenous control cues and on the environmental conditions experienced by seeds after their dispersal [44,45,46]. As part of this study, a one-year long seed burial experiment was run in order to analyse species’ ability to form a stable soil seed bank in their natural habitats. The results showed that both species produce a transient seed bank *sensu* Thompson [47,48]. In fact, seeds, after overwintering, had already germinated by the end of the following summer. 

Similarly, this behaviour was observed in seeds buried in the plant nursery, where the largest amount of germinations occurred by the end of the experiment in spring. 

Our results are consistent with Thompson’s assertions [45,49] that plants of stable habitats, such as mountain pastures, generally have seeds with a low persistence in the soil. This finding is also in agreement with the assumption of Arroyo et al. [50] that perennial species have a reduced tendency to form persistent seed banks compared to annual species. However, it should be pointed out that when attempting to correlate soil seed bank composition and dynamics of certain species with the features of their habitat (i.e., altitudinal gradients), contrasting results were obtained (see for example [46,51]). 

#### 3.2.2. Seed in the Soil in Relation to Different Microclimatic Conditions

Microclimatic conditions may play a greater role than altitude at the time of seed dispersal when seeds have the chance to either germinate or to form a soil seed bank [52]. Our climatic data showed a cold period with a *Ti* < 5 °C and monthly GDDs that did not exceed 200 °C during November 2015–March 2016 (Supplementary file S2) for both sites. Considering the temperature increase predicted to occur by the end of the 21st century for midlatitude mountains in Europe [53], the length of the snow cover on the mountain tops will almost certainly decline. A shorter duration of stable, low temperatures and a loss of the sheltering effect of a snow cover [6] would lead to an increased seedling mortality, which would in turn have substantial implications on the survival of the *Rhaponticum* species.

As a consequence of the thermal regime documented at the GDC site and considering the positive response to the duration of the cold period shown by the *R. bicknellii* seeds, we think that a reduction of the snow cover would have more severe and detrimental effects at GDC than at the PDV site. However, considering the low seed germination percentages and tendency to germinate at warmer temperatures, there is also a greater risk of seedling loss following drought stress during the summer for *R. scariosum*.

A shorter cold period would have an overall negative impact on the already very low annual rates of seedling recruitment observed in the two alpine *Rhaponticum* at both sites (Carasso, data not shown). A high seed mortality in the soil seed bank is documented for mountain environments [46], and this was explained by the extreme climatic variability of these habitats. Venn et al. [54] documented that seedlings in the Australian vegetation of the mountain summits often responded to the local conditions of their microsites and immediate surroundings. The soil wilting point and the local influences of soil moisture and plant litter were found to be among the factors that contributed the most to new seedling emergence. Hence, more detailed studies of the microclimatic conditions, at the soil level, in the natural habitats and adjacent areas are necessary to detect the presence of safe sites [55] and to help explain the discrepancy we found between seedling recruitment and the results of the seed burial experiments. Experiments that expose seeds to soil temperatures that mimic the current and projected alpine seasonal cycles could also help further disentangle the germination strategies of the studied species.

### 3.3. Microclimatic Conditions at the Growing Sites and in the Nursery

In this study we demonstrated the importance of interpreting germination responses in alpine species in the context of the typical bioclimatic characteristics of the growing sites. Our results confirmed the hypothesis that the different thermoclimatic conditions in the field resulted in different germination strategies in the two species. Although we tested only two populations, we assumed the response of their seeds as representative of the two species given their narrow geographic distribution. According to the features of the orotemperate thermoclimatic belt, seed germination (and presumably seedling development, too) in *R. scariosum* is most likely postponed until warmer air and soil conditions prevail. Under these circumstances, the seeds would require a warm stratification after the cold period, or more simply warmer temperatures to germinate. 

On the other hand, the supratemperate *R. bicknellii*, which, under the influence of a Mediterranean climate, experiences much warmer and drier conditions during the growing season, seem to adopt a drought-avoiding germination strategy to anticipate the occurrence of seed germination immediately after the snowmelt.

## 4. Materials and Methods

### 4.1. Species Taxonomy, Distribution and Study Sites

The genus *Rhaponticum* Vaill. (= *Stemmacantha* Cass.; cf. [56]) (Asteraceae) includes 25 species [57]. Those representatives of the genus which are endemic to the western Alps are treated in different ways by various authors. In this paper, we adopted the typification suggested by [27] and identified two species, *Rhaponticum scariosum* Lam. and *Rhaponticum bicknellii* (Briq.) Banfi, Galasso & Soldano (Table S1). Both species are protected under the Regional Piedmont Law 32/82. *R*. *scariosum* is a Least Concern species in the IUCN Red List of Threatened Species ver. 3.1 [58].

*R. scariosum* achenes were collected from a population in Alta Valle Stura di Demonte, Prati del Vallone (Pietraporzio, Piedmont, Italy) at 1915 m a.s.l. (Figure 1; Table 4). 

The typical substrate in this area consists of rocky ledges and thin elements on siliceous screes but also of rocky slopes that are always under full light at an altitude of up to 2500 m a.s.l. [25]. *R. bicknellii* seeds were collected in Valle Tanaro, Gola della Chiusetta (Briga alta, Piedmont, Italy) at 1840 m a.s.l. (Figure 1; Table 4). This population is located on a south facing, grassy slope. The species is normally found in mesophile microdepressions [25], and only a few small populations reach altitudes of up to 2000 m a.s.l.

According to the floristic classification system of the vegetation of Europe [59], *R. scariosum* belongs to the alpine and subalpine silicicolous swards of the mountain ranges (TRI-02 *Caricetalia curvulae* Br.-Bl.). Plant communities of *R. bicknellii* belong to both the mesoxerophytic grasslands of Western and Central Europe (FES-01 *Brachypodietalia pinnati* Korneck 1974 nom. conserv. propos.) and to the alpine and subalpine calicicolous grasslands of the nemoral mountain ranges of central Europe (SES-01 *Seslerietalia caeruleae* Br.-Bl.).

### 4.2. Study 1—Microclimatic Conditions at the Growing Sites and in the Plant Nursery

Microclimatic data were recorded at the two study sites to decide upon the incubation temperatures for the germination tests in the lab and to calculate climatic parameters (see below). The latter were used to help interpret results of the germination tests in accordance with the climate of the study areas and of the thermoclimatic belts at the growing sites. Temperatures at the soil surface and relative humidity percentages were recorded daily for the 2015–2016 period using two data loggers (Tiny Tag, Gemini, Chichester, UK). Precipitation data (shown as monthly precipitation and number of rainy days with precipitation > 1 mm/day) come from two meteorological stations in Alta Valle Stura di Demonte next to Prati del Vallone (Argentera, Cuneo, code 298) and in Valle Tanaro next to Gola della Chiusetta (Upega, Briga Alta, Cuneo, code 308). These weather stations belong to the ARPA-Piemonte weather monitoring network.

In the regional nursery temperature at soil surface and relative humidity percentages were recorded for the period October 2018–June 2019 using a data logger (same model as above) left 9 months at the soil level.

### 4.3. Study 2—Conversion of Temperature Data into Different Climatic Parameters

Soil temperature recordings (in °C) and the relative humidity (RH in %) at the two study sites of GDC and PDV were tabulated as weekly means and indicated as WOY (week of the year) for the 2015–2016 period. The soil temperature thresholds are plotted in Figure 2 as the mean daily temperatures (°C).

Local temperature records of GDC and PDV were used to calculate two temperature parameters: yearly positive temperatures (*Tp*) and growing degree-days (*GDDs*). Positive temperatures were calculated as the sum of the mean temperature of any month of the year (*Ti*) above 0 °C [24]. *Tp* was used because it is representative of the local thermotypes. The latter are bioclimatic indices that are normally designated according to: (1) the compensated thermicity index (*Itc*), which evaluates the intensity of the cold during the coldest month as a limiting factor for plant development, and (2) the *Tp*, which was calculated as above. However, in cold mountain habitats (where *Itc* < 120; [60]), it is more relevant and precise to refer only to the *Tp* values. Thermoclimatic belts of the two species were assessed by mapping the two study areas (GDC and PDV) on the WBCS [24]. The resulting theoretical *Tp* values, according to Rivas-Martínez et al. [23], are reported in the manuscript as a mean of comparison with the locally estimated *Tp* values.

In addition, the temperature data were used to calculate the monthly growing degree-days (GDDs) [33]. *GDDs* are used as a measure of the heat energy (in °C) accumulated in a given geographic area and available for growth at any phenotypic stage of the life cycle of a plant. GDDs were calculated according to the formula:

*GDDs* = [(maximum daily temperature + minimum daily temperature)/2] − base temperature

Negative values of accumulated degrees were displayed as zero. The daily *GDDs* were then calculated for each month and for the whole year (Yearly *GDDs*; August 2015–July 2016). Following Venn and Morgan [46], we chose 0 °C as a conservative base growing temperature (= the temperature above which plant cells show metabolic functions) that is suitable for most alpine plants.

### 4.4. Study 3—Germination Experiments under Controlled Conditions in the Lab

#### 4.4.1. Seed Collection and Preparation

Seeds of both species were collected in August 2015, at the time of natural dispersal, from 14 individuals belonging to two macropopulations consisting of several small groups scattered throughout the two study sites. Seeds of *R. bicknellii* were collected at SIC IT 1160057 within the Parco Naturale del Marguareis (Alta Valle Tanaro, CN) and seeds of *R. scariosum* were collected at SIC IT 1160021 Gruppo del Tenibres (Alta Valle Stura, CN). The average number of fruiting plants per group was 47 for *R. bicknellii* and 22 for *R scariosum*. The seeds were immediately brought to the laboratory and stored under standard drying conditions (15 °C, 15% RH) for two weeks. The initial viability of the seeds was checked through a Tetrazolium (2,3,5-triphenyltetrazolium chloride) test (TZ test). All tested seeds were found to be viable.

#### 4.4.2. Germination Tests

For germination tests of both species, we used four different cold stratification durations and three different germination temperatures. Replicates were incubated in the dark (wrapped in aluminium foil) for about five months and organized as shown in Table 5. 

Seeds were sown on a 1% w/v agar medium in 90 mm diameter sterile Petri dishes sealed with paraffin tape and exposed to 12 different treatments, with three replicates of 20 seeds each. The temperatures (10 °C, 15 °C and 20 °C) were chosen based on the temperatures recorded near the growing sites during the previous years (data not shown). Considering different winter lengths at the two localities, the seeds were subjected to a cold stratification at 5 °C [11,61] for 0, 30, 60 and 90 days. After the cold stratification, the seeds were transferred to a germination chamber (Sanyo MIR-154) and checked for germination twice a week. Seeds were considered germinated when their root apex protruded by more than 2 mm. The viability of those seeds which had not germinated after 157 days was checked by means of the TZ test. A cut-test identified empty seeds which were excluded from the further analysis.

### 4.5. Study 4—Seed Burial Experiments at the Study Sites and in the Plant Nursery

Seed burial experiments were carried out at the two study sites to assess whether the two species were able to form a soil seed bank. The experiments were started at the time of natural seed dispersal in August 2015 and lasted one year (Supplementary files S7 and S8). Seeds were collected from 12 plants of each species. For each species, five replicates of 20 fresh dispersal units (complete achenes including the pappus) were placed into nylon mesh sachets (mesh size 0.5 mm) that were half-filled with sieved top-soil taken from underneath the adult plants. Close to the mother plants, the sachets were buried in the soil at a depth of about 5 cm and fixed with stainless-steel rods. Each burial site contained four sachets, which were placed at the vertices of a meter-square plot plus a fifth one, which was placed in the centre. All sachets were dug up after one year and moved to the laboratory, where the empty and intact seeds and plant remnants were counted.

In 2018, a further seed burial experiment was prepared in the regional nursery “Gambarello” (Chiusa di Pesio, Cuneo) in order to check seed monthly germinations of the two species placed at the same environmental conditions. For each species, nine replicates of 20 dried seeds (from the same accessions of 2015) were placed into nylon mesh sachets (mesh size 0.5 mm) that were half-filled with a mix of peat, vermiculite and agriperlite (proportions 70:15:15). The bags were buried at a depth of about 5 cm in a plastic plateau filled with the same soil mix. The sachets were recovered on a monthly base, starting from October to June of the following year and moved to the laboratory where the germinated, empty and intact sees were counted. The experiment lasted 240 days (Supplementary file S9). 

### 4.6. Statistical Analysis

The influence of the species, of the duration of cold stratification and of the incubation temperature on seed germination were evaluated by means of Generalized Linear Models (GLM). The binomial error structure and logit link function were chosen in GLM. 

The final germination was considered as the response variable, whereas the species (taken as a two-level categorical variable), the duration of cold stratification (taken as a continuous variable: 0, 30, 60 and 90 days), the incubation temperature (taken as a continuous variable: 10 °C, 15 °C and 20 °C) and their two-way and three-way interactions were the fixed effects. In addition, species-specific GLMs were performed with final germination values as the response variables, and the duration of the cold stratification period, the incubation temperature and their interaction as explanatory variables. In order to gain a better understanding of the influence of the incubation temperature and the duration of the cold stratification period, other GLMs were performed at a species level. The best model was selected by comparing the residual deviance and, in the absence of overdispersion, the Akaike information criterion (AIC). A binomial error structure and logit link functions were chosen in the GLM analysis for the *R. scariosum* seeds, while a quasibinomial error structure was used for the *R. bicknellii* seeds, due to overdispersion. The minimal adequate model was obtained for each species by excluding nonsignificant terms, according to Crawley [62]. In addition, to study the effects of higher incubation temperatures on the speed of seed germination, the mean time to germinate (MTG) was calculated as

MTG = *Ʃ^n^_i_, n_i_ t_i_ / N*

where *n_i_* is the number of seeds that germinated within consecutive intervals of time, *t_i_* is the time between the beginning of the test and the end of a measurement interval and *N* is the total number of seeds that germinated. The MTG was only calculated for the fresh seeds using the day of sowing as the initial time, this having been calculated after transferring the seeds to environments with 10 °C, 15 °C and 20 °C. The influence of the species, incubation temperature and their two-way interactions on the MTG were analysed by means of GLM, with a gamma error structure and inverse link function. All analyses were performed using R software (version 3.3.1, R Core Team 2016).

## 5. Conclusions

Climate warming reduces suitable ranges of alpine species and alters their community compositions. These changes also affect the early life stages of a plant. Our study poses the question of how *R. scariosum* and *R. bicknellii* populations will adapt to future changes in their alpine habitat, considering that they do not form a persistent soil seed bank which could otherwise function as a buffer against the extinction of small and isolated populations. 

We provide evidence of significant differences in germination strategies between the two endemic and closely related *Rhaponticum* species, which can be interpreted from a biogeographical and bioclimatic perspective. The results of the germination tests supported our hypothesis that seeds of *R*. *scariosum* and *R. bicknellii* are physiologically dormant at the time of dispersal. However, only in *R. bicknellii* the seed dormancy alleviation depends on the duration of the cold period. This finding seems to indicate that at supratemperate thermotypes, species are more vulnerable to climate warming than at orotemperate thermotypes.

Germination changes in response to several other environmental factors in addition to temperature. In climatically variable and unpredictable alpine environments, the interplay between temperature and rainfall at the time of germination may exert a selective pressure on germination timing. We showed that the need for a long period of cold stratification and the ability to germinate at low temperatures are two key features of *R. bicknellii*. Field climatic data at the GDC site showed that its seedlings experienced a strong drought pressure during late spring. A “cold” germination strategy in this species was supported by air temperature and precipitation trends at the site and it may be correlated to the supratemperate thermal regime of this geographic area.

In contrast, *R. scariosum* only requires a short period of cold stratification at lower average air temperatures and an evenly distributed rainfall that is typical of the orotemperate thermal regime, coupled with an inability to germinate at low temperatures. This postpones seedling emergence to late spring and prevents seed germination at low temperatures.

Environmental variations at a local scale can influence germination timing in nature and this leads to differences in the dormancy release, germination rate and timing of seedling emergence for the different species and different populations. This article shows the importance of interpreting germination data from a bioclimatic perspective.

## Figures and Tables

**Table 1:** Results of the GLM analysis for the responses of the final seed germination in relation to the different incubation temperatures (T), stratification periods (Str), species (Sp) and their interactions. Nonsignificant interactions were excluded during model selection and are not reported. The models were performed with a binomial error and logit link function for the final germination percentages.

Factor	Estimate	*SE*	*z*-Value	*p*
Intercept	−3.228926	0.325025	−9.934	<2 × 10^−16^ ***
Cold stratification (Str)	0.004309	0.003156	1.365	0.17218
Incubation temperature (T)	0.083810	0.016694	5.020	5.16 × 10^−7^ ***
*R. bicknellii*	0.201804	0.247832	0.814	0.41549
Str x *R. bicknellii*	0.011759	0.004139	2.841	0.00449 **

Estimated coefficient, the standard error (*SE*), *z*-value and *p*-value are shown. Significant values are indicated as *** (*p* < 0.001), ** (*p* < 0.01) and * (*p* < 0.05).

**Table 2:** Results of the GLM analysis to establish the responses of the final seed germination in relation to the different incubation temperatures (T) and stratification periods (Str) for the *R. scariosum* seeds. The models were performed with a binomial error and logit link function for the final germination percentages.

Factor	Estimate	*SE*	*z*-Value	*p*
Intercept	−2.9257	0.4524	−6.467	1.00 × 10^−10^ ***
Cold stratification (Str)	0.0043	0.0032	1.350	0.177
Incubation temperature (T)	0.0649	0.0262	2.484	0.013 *

The estimated coefficient, standard error (*SE*), *z*-value and *p*-value are shown. Significant values are indicated as *** (*p* < 0.001), ** (*p* < 0.01) and * (*p* < 0.05).

**Table 3:** GLM analysis results for the responses of the seed final germination in relation to the different incubation temperatures (T) and stratification periods (Str) for the *R. bicknellii* seeds. The models were performed with a quasibinomial error and logit link function for the final germination.

Factor	Estimate	*SE*	*t*-Value	*p*
Intercept	−3.2281	0.3883	−8.314	4.65 × 10^−16^ ***
Cold stratification (Str)	0.0162	0.0027	6.004	3.06 × 10^−9^ ***
Incubation temperature (T)	0.0963	0.0212	4.432	1.08 × 10^−5^ ***

The estimated coefficient, standard error (*SE*), *t*-value and *p*-value are shown. Significant values are indicated as *** (*p* < 0.001), ** (*p* < 0.01) and * (*p* < 0.05).

**Table 4:** Locations and characteristics of the habitats of the two Rhaponticum species.

Species	Study Areas (Acronyms)	Habitat Code	SIC Code ^b^	Geology	Altitude (m a.s.l.)	Aspect	Thermoclimatic Belts ^c^
*R. scariosum*	PDV ^a^	Alpine pastures: 6150	IT 1160021	Siliceous rocks	1915	South-East	Orotemperate
*R. bicknellii*	GDC ^a^	Alpine pastures: 6170 *	IT 1160057	Calcareous rocks	1840	South	Supratemperate

^a^ PDV = Prati del Vallone (CN); GDC = Gola della Chiusetta (CN); ^b^ Natura 2000 network (Sindaco et al. 2009); ^c^ Defined according to Rivas-Martínez et al. [31]. * *p* < 0.05.

**Table 5:** Experimental design of the germination tests conducted in the laboratory. Cold stratification at 5 °C was followed by three different incubation temperatures.

Treatments in the Dark	Cold Stratification Period (in Days)				Incubation Temperatures (°C)		
	0	30	60	90	10	15	20
1	X				X		
2		X			X		
3			X		X		
4				X	X		
5	X					X	
6		X				X	
7			X			X	
8				X		X	
9	X						X
10		X					X
11			X				X
12				X			X

## Supplementary Materials

The following are available online at https://www.mdpi.com/2223-7747/9/6/708/s1. S1—Taxonomic overview of the *Rhaponticum* species. S2—Weekly soil temperatures and relative humidity for the whole year. S3—Weekly soil temperatures and relative humidity for the eight months in the nursery. S4—Climatic parameters. S5—Analysis of deviance tables. S6—Final germination for different cold stratification periods. S7—Outline of the seed burial experiments in the field. S8—Weekly soil T and RH during seed dispersal. S9—Outline of the seed burial experiments in nursery.
