# Supplementary material for: Comparative Germination Ecology of Two Endemic Rhaponticum Species (Asteraceae) in Different Climatic Zones of the Ligurian and Maritime Alps (Piedmont, Italy)
Source: Plants (Basel). 2020 Jun 2;9(6):708. doi: 10.3390/plants9060708 (PMC7356568; doi:10.3390/plants9060708)
Supplement: Supplementary file 1 [file plants-09-00708-s001.zip › plants-791857-supplementary-2/S_2 Weekly soil T and RHy for whole year.docx]

**SUPPLEMENTARY MATERIALS S2**

**Comparative germination ecology of two endemic *Rhaponticum* species (*Asteraceae*) in different climatic zones of the Ligurian and Maritime Alps (Piedmont, Italy)**

Plants

**Valentina Carasso^1, *^, Marco Mucciarelli^2^, Francesco Dovana^2^, Jonas V Müller^3^**

^1^Centro Regionale Biodiversità Vegetale, Ente di gestione delle Aree Protette delle Alpi Marittime, Via S. Anna, 34, 12013 Chiusa di Pesio, Italy; valentina.carasso@virgilio.it

^2^Università di Torino, Department of Life Sciences and Systems Biology, Viale P.A. Mattioli, 25, 10125 Torino, Italy; marco.mucciarelli@unito.it; francescodovana@libero.it

^3^Royal Botanic Gardens Kew, Millennium Seed Bank, Conservation Science, Wakehurst Place, Ardingly, West Sussex, RH17 6TN, United Kingdom; j.mueller@kew.org

*Correspondence: valentina.carasso@virgilio.it

**Table S2**. Weekly average soil temperature (±s.e.) expressed in °C and weekly average relative humidity (%) at the two sites of the seed burial experiments (GDC and PDV) for the whole year.

| **Year** | **Month** | **WOY** | **Soil Temperature (°C)** | | **Relative Humidity (%)** | |
| --- | --- | --- | --- | --- | --- | --- |
|  |  |  | **GDC** | **PDV** | **GDC** | **PDV** |
| 2015 | Jul | 29 | 23.06 ± 0.66 | 19.82 ± 2.1 | 51.15 ± 1.75 | 67.11 ± 6.15 |
|  | Jul | 30 | 20.29 ± 0.57 | 16.42 ± 0.29 | 57.81 ± 3.06 | 56.11 ± 6.92 |
|  | Jul-Aug | 31 | 18.67 ± 0.88 | 14.31 ± 0.99 | 64.41 ± 3.79 | 59.61 ± 9.14 |
|  | Aug | 32 | 19.14 ± 1.23 | 14.92±1.01 | 64.74±5.53 | 53.42±9.1 |
|  | Aug | 33 | 14.35 ± 0.89 | 11.22 ± 0.81 | 75.33 ± 3.86 | 78.48 ± 2.69 |
|  | Aug | 34 | 13.49 ± 0.49 | 11.05 ± 0.47 | 75.68 ± 3.38 | 51.6 ± 13.1 |
|  | Aug-Sept | 35 | 16.61 ± 0.42 | 14.26 ± 0.47 | 66.52 ± 3.27 | 75.03 ± 1.36 |
|  | Sept | 36 | 12.64 ± 0.25 | 9.3 ± 0.44 | 69.58 ± 0.98 | 73.45 ± 4.68 |
|  | Sept | 37 | 10.53 ± 0.37 | 8.36 ± 0.41 | 77.46 ± 10.19 | 38.26 ± 6.94 |
|  | Sept | 38 | 10.88±0.55 | 9.07±0.53 | 74.98 ± 2.94 | 68.62 ± 1.6 |
|  | Sept | 39 | 8.27 ± 1 | 5.75 ± 1.22 | 80.8 ± 2.29 | 57.21 ± 6.45 |
|  | Oct | 40 | 6.55±1.27 | 4.94±1.61 | 91.97±3.71 | 79.79±3.73 |
|  | Oct | 41 | 7.14 ± 0.34 | 5.84 ± 0.44 | 94.66 ± 1.84 | 47.63 ± 9.61 |
|  | Oct | 42 | 4.07 ± 0.57 | 2.82 ± 0.73 | 91.97 ± 3.71 | 79.79 ± 3.73 |
|  | Oct | 43 | 7.18 ± 0.53 | 6.01 ± 1.34 | 60 ± 2.22 | 48.21 ± 8.52 |
|  | Oct-Nov | 44 | 5.14±0.29 | 3.06±0.53 | 76.45±6.9 | 64.29±6.22 |
|  | Nov | 45 | 8.63 ± 0.77 | 9.75 ± 1.09 | 50.65 ± 8.25 | 40.45 ± 7.23 |
|  | Nov | 46 | 7.9 ± 0.29 | 8.97 ± 0.35 | 46.95 ± 3.47 | 31.39 ± 2.82 |
|  | Nov | 47 | 2.43 ± 1.74 | -0.71 ± 2.36 | 61.84 ± 8.57 | 56.75 ± 8.04 |
|  | Nov-Dec | 48 | 1.43 ± 1.13 | 1.33 ± 1.75 | 56.25 ± 8.64 | 34.59 ± 5.8 |
|  | Dec | 49 | 2.22 ± 0.36 | 2.03 ± 0.86 | 77.46 ± 10.03 | 63.8 ± 11.53 |
|  | Dec | 50 | 1.68 ± 0.53 | 2.21 ± 0.93 | 63.63 ± 9.84 | 42.15 ± 7.13 |
|  | Dec | 51 | 2.33 ± 0.4 | 2.56 ± 0.58 | 77 ± 4.73 | 53.47 ± 7.71 |
|  | Dec | 52 | 1.55 ± 0.21 | 2.14 ± 0.77 | 59.7 ± 7.05 | 39.74 ± 6.04 |
| 2015-2016 | Dec-Jan | 53 | -0.22 ± 0.43 | -1.75 ± 0.62 | 81.34 ± 3.56 | 71.15 ± 4.44 |
| 2016 | Jan | 1 | 0.54 ± 0.78 | -0.49 ± 1.46 | 72.17 ± 6.92 | 65.39 ± 4.32 |
|  | Jan | 2 | -4.67 ± 0.64 | -7.85 ± 0.96 | 70.9 ± 5.72 | 64.18 ± 5.3 |
|  | Jan | 3 | 1.23 ± 1.27 | 1.33 ± 1.63 | 54.53 ± 7.25 | 47.05 ± 2.22 |
|  | Jan | 4 | 4.65 ± 0.97 | 4.73 ± 1.22 | 53.68 ± 5.12 | 46.03 ± 6.76 |
|  | Jan-Feb | 5 | 0.76 ± 0.47 | -0.74 ± 1.29 | 21.62 ± 10.33 | 52.4 ± 4.54 |
|  | Feb | 6 | 1.06 ± 0.08 | -3.2 ± 0.7 | 1.56 ± 1.39 | 58.11 ± 6.76 |
|  | Feb | 7 | 1.01 ± 0.1 | 0.9 ± 1.76 | 15.42 ± 7.49 | 55.19 ± 5.39 |
|  |  |  |  |  |  |  |
|  | Feb | 8 | 0.14 ± 0.16 | -1.51 ± 0.83 | 13.54 ± 5.46 | 48.54 ± 9.71 |
|  | Feb-Mar | 9 | **0.55 ± 0.02** | -4.93 ± 0.55 | **6.77 ± 0.05** | 63.08 ± 3.13 |
|  | Mar | 10 | **0.62 ± 0.01** | -2.04 ± 0.72 | **7.72 ± 0.16** | 80.52 ± 3.51 |
|  | Mar | 11 | **0.67 ± 0.06** | 0.15 ± 0.89 | **8.13 ± 0.35** | 72.36 ± 4.89 |
|  | Mar | 12 | **0.53 ± 0.04** | 3.58 ± 0.43 | **9.22 ± 0.2** | 56.79 ± 2.93 |
|  | Mar-Apr | 13 | **0.37 ± 0.02** | 2.97 ± 0.55 | **9.68 ± 0.08** | 27.59 ± 10.33 |
|  | Apr | 14 | 0.86 ± 0.27 | 4.51 ± 0.56 | 22.08 ± 13.18 | 66.17 ± 3.1 |
|  | Apr | 15 | 6.4 ± 0.8 | 5.62 ± 0.73 | 67.64 ± 8.32 | 52.19 ± 4.01 |
|  | Apr | 16 | 5.96 ± 0.69 | 3.65 ± 0.44 | 73.98 ± 5.5 | 50.96 ± 6 |
|  | Apr | 17 | 5.68 ± 1.01 | 3.7 ± 1.06 | 74.37 ± 5.99 | 67.92 ± 7.29 |
|  | May | 18 | 6.27 ± 0.3 | 3.78 ± 0.41 | 64.1 ± 8.58 | 57.52 ± 9.2 |
|  | May | 19 | 7.22 ± 0.36 | 6.52 ± 0.36 | 84.83 ± 1.17 | 72.91 ± 3.36 |
|  | May | 20 | 11 ± 0.78 | 8.23 ± 1.23 | 61.48 ± 3.23 | 56.54 ± 2.12 |
|  | May | 21 | 11.66 ± 1.19 | 9.14 ± 1.26 | 80.23 ± 4.18 | 74.37 ± 5.76 |
|  | May-Jun | 22 | 11.23 ± 0.5 | 9.39 ± 0.53 | 89.16 ± 2.44 | 88.69 ± 2.25 |
|  | Jun | 23 | 13.29 ± 0.38 | 11.07 ± 0.41 | 79.09 ± 2.46 | 66.9 ± 4.02 |
|  | Jun | 24 | 13.18 ± 1.29 | 9.58 ± 1.32 | 75.71 ± 3.58 | 74.31 ± 4.96 |
|  | Jun | 25 | 18.66 ± 0.46 | 15.29 ± 0.46 | 67.83 ± 1.66 | 69.57 ± 4.77 |
|  | Jun-Jul | 26 | 18.33 ± 0.57 | 14.77 ± 0.36 | 74.26 ± 2.43 | 71.77 ±2.44 |
|  | Jul | 27 | 19.91 ± 0.69 | 15.89 ± 0.58 | 69.18 ± 3.33 | 68.91 ± 5.07 |
|  | Jul | 28 | 19.13 ± 1.42 | 13.42 ± 1.76 | 56.09 ± 2.34 | 61.82 ± 4.73 |

PDV = Prati del Vallone (CN); GDC = Gola delle Chiusette (CN). Different styling indicate the significant events during the winter: temperatures <2 °C without snow cover are underlined; temperatures <2 °C and relative humidity <10% with snow cover are in bold.
