# Supplementary material for: Comparative Germination Ecology of Two Endemic Rhaponticum Species (Asteraceae) in Different Climatic Zones of the Ligurian and Maritime Alps (Piedmont, Italy)
Source: Plants (Basel). 2020 Jun 2;9(6):708. doi: 10.3390/plants9060708 (PMC7356568; doi:10.3390/plants9060708)
Supplement: Supplementary file 1 [file plants-09-00708-s001.zip › plants-791857-supplementary-2/S_3 Weekly soil T and RH for the 8 months in nursery.docx]

**SUPPLEMENTARY MATERIALS S3**

**Comparative germination ecology of two endemic *Rhaponticum* species (*Asteraceae*) in different climatic zones of the Ligurian and Maritime Alps (Piedmont, Italy)**

Plants

**Valentina Carasso^1, *^, Marco Mucciarelli^2^, Francesco Dovana^2^, Jonas V Müller^3^**

^1^Centro Regionale Biodiversità Vegetale, Ente di gestione delle Aree Protette delle Alpi Marittime, Via S. Anna, 34, 12013 Chiusa di Pesio, Italy; valentina.carasso@virgilio.it

^2^Università di Torino, Department of Life Sciences and Systems Biology, Viale P.A. Mattioli, 25, 10125 Torino, Italy; marco.mucciarelli@unito.it; francescodovana@libero.it

^3^Royal Botanic Gardens Kew, Millennium Seed Bank, Conservation Science, Wakehurst Place, Ardingly, West Sussex, RH17 6TN, United Kingdom; j.mueller@kew.org

*Correspondence: valentina.carasso@virgilio.it

**Table S3**. Weekly average soil temperature (±s.e.) expressed in °C and weekly average relative humidity (%) for the seed burial experiments at the Gambarello nursery (Chiusa di Pesio) for 8 months.

| **Year** | **Month** | **WOY** | **Soil Temperature (°C)** | **Relative Humidity (%)** |
| --- | --- | --- | --- | --- |
|  | Oct | 41 | 14.23 | 58.87 |
|  | Oct | 42 | 13.09 | 58.07 |
|  | Oct | 43 | 11.59 | 68.51 |
|  | Oct-Nov | 44 | 9.10 | 29.07 |
|  | Nov | 45 | 11.41 | 19.83 |
|  | Nov | 46 | 8.60 | 56.36 |
|  | Nov | 47 | 3.99 | 37.47 |
|  | Nov-Dec | 48 | 3.61 | 53.61 |
|  | Dec | 49 | 5.20 | 63.31 |
|  | Dec | 50 | 0.91 | 81.77 |
|  | Dec | 51 | 1.63 | 43.80 |
|  | Dec | 52 | 3.66 | 76.29 |
| 2018-2019 | Dec-Jan | 0 | 1.73 | 73.87 |
| 2019 | Jan | 1 | 0.81 | 70.43 |
|  | Jan | 2 | 1.66 | 74.73 |
|  | Jan | 3 | -1.70 | 70.21 |
|  | Jan | 4 | -0.51 | 50.49 |
|  | Jan-Feb | 5 | 1.60 | 83.29 |
|  | Feb | 6 | 3.49 | 74.64 |
|  | Feb | 7 | 5.19 | 79.14 |
|  | Feb | 8 | 6.97 | 51.53 |
|  | Feb-Mar | 9 | 7.86 | 63.41 |
|  | Mar | 10 | 9.03 | 53.34 |
|  | Mar | 11 | 9.04 | 46.60 |
|  | Mar | 12 | 8.70 | 54.43 |
|  | Mar-Apr | 13 | 9.27 | 44.39 |
|  | Apr | 14 | 8.70 | 54.51 |
|  | Apr | 15 | 13.19 | 67.33 |
|  | Apr | 16 | 11.01 | 46.50 |
|  | Apr | 17 | 12.83 | 56.65 |
|  | May | 18 | 14.23 | 64.71 |
|  | May | 19 | 12.37 | 45.79 |
|  | May | 20 | 16.03 | 49.83 |
|  | May | 21 | 18.93 | 50.80 |
|  | May-Jun | 22 | 19.91 | 72.03 |
|  | Jun | 23 | 21.36 | 71.19 |
