# Supplementary material for: Comparative Germination Ecology of Two Endemic Rhaponticum Species (Asteraceae) in Different Climatic Zones of the Ligurian and Maritime Alps (Piedmont, Italy)
Source: Plants (Basel). 2020 Jun 2;9(6):708. doi: 10.3390/plants9060708 (PMC7356568; doi:10.3390/plants9060708)
Supplement: Supplementary file 1 [file plants-09-00708-s001.zip › plants-791857-supplementary-2/S_4 Climatic Parameters.docx]

**SUPPLEMENTARY MATERIALS S4**

**Comparative germination ecology of two endemic *Rhaponticum* species (*Asteraceae*) in different climatic zones of the Ligurian and Maritime Alps (Piedmont, Italy)**

Plants

**Valentina Carasso^1, *^, Marco Mucciarelli^2^, Francesco Dovana^2^, Jonas V Müller^3^**

^1^Centro Regionale Biodiversità Vegetale, Ente di gestione delle Aree Protette delle Alpi Marittime, Via S. Anna, 34, 12013 Chiusa di Pesio, Italy; valentina.carasso@virgilio.it

^2^Università di Torino, Department of Life Sciences and Systems Biology, Viale P.A. Mattioli, 25, 10125 Torino, Italy; marco.mucciarelli@unito.it; francescodovana@libero.it

^3^Royal Botanic Gardens Kew, Millennium Seed Bank, Conservation Science, Wakehurst Place, Ardingly, West Sussex, RH17 6TN, United Kingdom; j.mueller@kew.org

*Correspondence: valentina.carasso@virgilio.it

**Supplementary file S4 - Climatic parameters**

**Table S4** Temperature parameters at PDV = Prati del Vallone (Cuneo) and GDC = Gola delle Chiusette (Cuneo). Mean temperature (*Ti*) (°C) from August 2015 to July 2016 calculated as Ti = ((T*max*+T*min*)/2) for the months with average temperatures >0 °C (see also Fig. 7). Yearly positive temperatures (*Tp*) calculated as the sum of the monthly mean temperatures and expressed in tenths of degrees. Accumulated monthly growing degree-days (*GDDs*) (see also Fig. 8) and their sum (Yearly *GDDs*) (see [33] and climatic data in the manuscript for calculation). In bold data for the growing season at these elevations (i.e., months with average temperature ≥5 °C)

|  | Years  2015-2016 | PDV | GDC | PDV | GDC |
| --- | --- | --- | --- | --- | --- |
| # |  |  |  |  |  |
|  |  | *Ti* (°C) | *Ti* (°C) | *GDDs* (°C) | *GDDs* (°C) |
| 1 | August | **13.2** | **17.3** | **420.1** | **538.4** |
| 2 | September | **6.3** | **9.8** | **267.3** | **340.5** |
| 3 | October | **5.1** | **5.9** | **172.5** | **196.1** |
| 4 | November | 3.2 | 4.4 | 200.3 | 171.9 |
| 5 | December | 2.5 | 2.7 | 114.4 | 71.5 |
| 6 | January | 0.0 | 1.1 | 47.8 | 41.8 |
| 7 | February | 1.4 | 3.2 | 47.4 | 48.5 |
| 8 | March | 0.0 | 0.7 | 56.8 | 24.9 |
| 9 | April | **4.8** | **5.1** | **146.8** | **132.6** |
| 10 | May | **6.4** | **9.6** | **242.5** | **304.6** |
| 11 | June | **11.2** | **13.9** | **373.9** | **448.1** |
| 12 | July | **12.6** | **18.4** | **489.9** | **659.6** |
|  | *Tp* | 667.75 | 921.0 | - | - |
|  | Yearly *GDDs* | - | - | 2579.7 | 2978.4 |


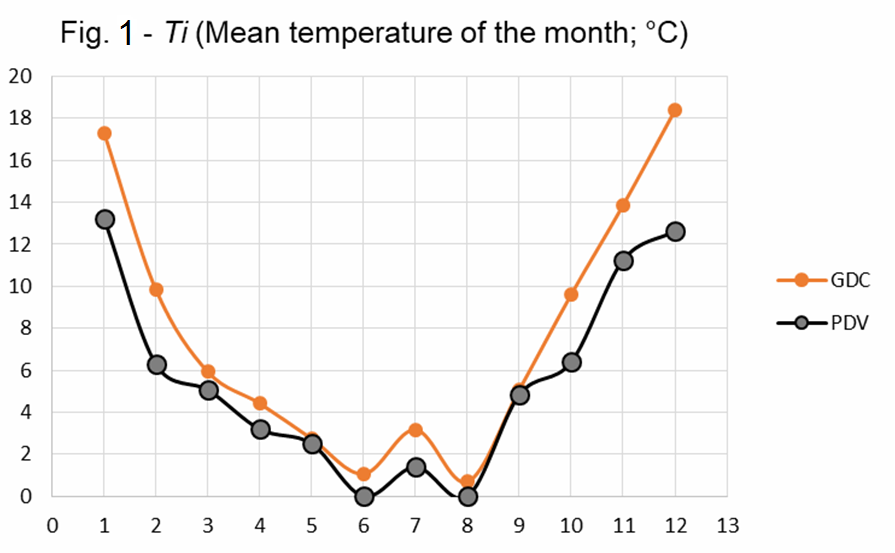


**Figure S1.** Ti (Mean temperature of the month; °C).


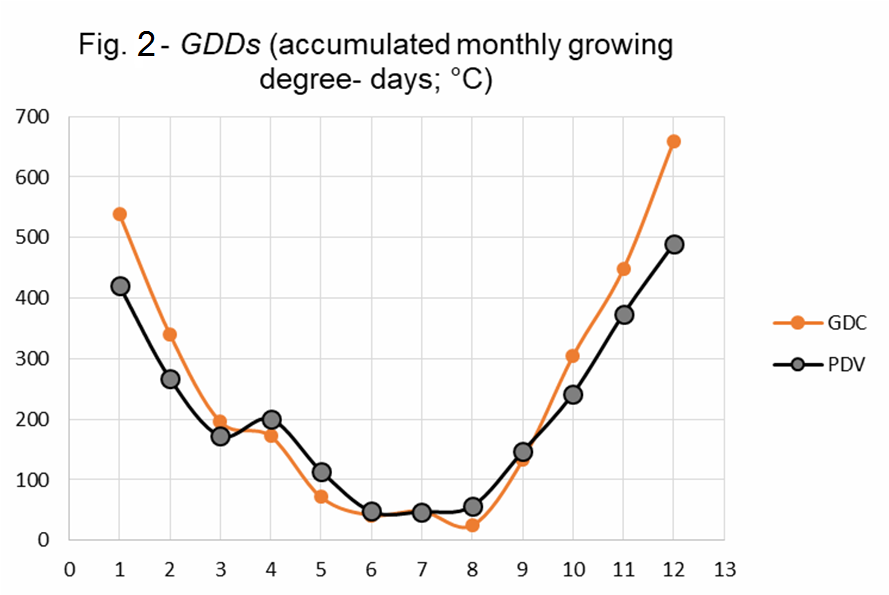


**Figure S2.** GDDs (accumulated nonthly growing degree-days; °C).

**Table S5** Monthly precipitations (mm) and monthly rainy days (precipitation >1 mm/day) for the growth period (i.e., months with average temperature ≥5 °C; see Table 1) (Fig. 9) at the two study sites; values are averages of a six year-period (2012/2017). Total precipitation (mm) and total rainy days for the growth period (April/October) per each year (Fig. 10). Climatic data for PDV and GDC sites are from the Argentera (CN) and Upega (Briga Alta, CN) ARPA-Piemonte meteorological stations, respectively.

|  | **Monthly precipitation** | | **Monthly rainy days** | |  | **Total precipitation** | | **Total raining days** | |
| --- | --- | --- | --- | --- | --- | --- | --- | --- | --- |
| *Month of the year* | PDV | GDC | PDV | GDC | *Years* | PDV | GDC | PDV | GDC |
| April (*n*=4) | 125.1 | 108.4 | 14.0 | 11.0 | 2012 | 751.0 | 641.2 | 71 | 55 |
| May (*n*=5) | 114.0 | 96.7 | 12.4 | 9.6 | 2013 | 725.6 | 721.4 | 74 | 65 |
| June | 79.9 | 61.1 | 9.8 | 8.7 | 2014 | 497.2 | 522.2 | 76 | 62 |
| July | 68.8 | 91.4 | 9.5 | 8.2 | 2015* | 587.4 | 323.4 | 52 | 42 |
| August | 37.9 | 48.8 | 5.5 | 4.7 | 2016* | 395.0 | 476.2 | 48 | 44 |
| September | 102.8 | 85.1 | 8.8 | 7.2 | 2017* | 246.2 | 359.0 | 39 | 36 |
| October (*n*=4) | 113.1 | 122.0 | 10.0 | 7.0 | − | − | − | − | − |

*n* = number of years available for the computation when different from six; * Data were not available for each month of the period (April/October), thus yearly precipitations and rainy days have been computed in order to be comparable among the two study sites but are not representative of that specific year.


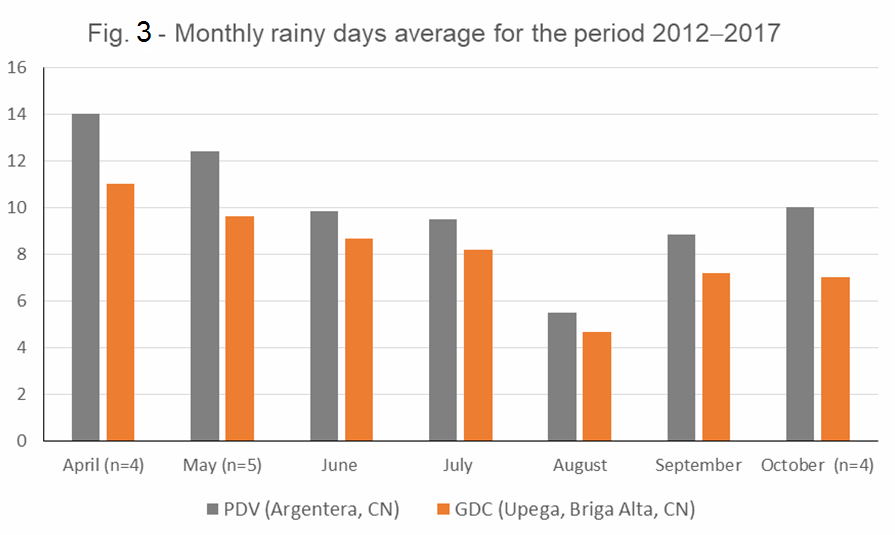


**Figure S3.** Monthly.rainy days average for the period 2012-2017.


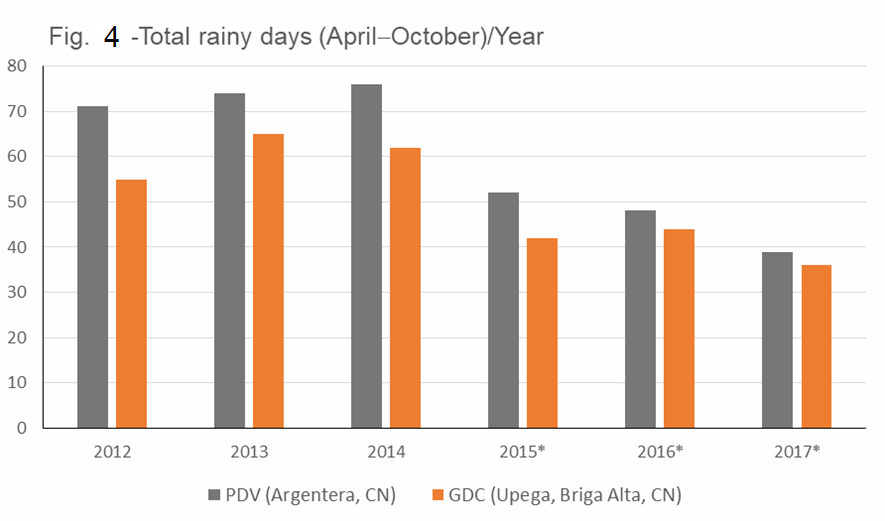


**Figure S4.** Total.rainy days (April-October)/Year.
