# Supplementary material for: Comparative Germination Ecology of Two Endemic Rhaponticum Species (Asteraceae) in Different Climatic Zones of the Ligurian and Maritime Alps (Piedmont, Italy)
Source: Plants (Basel). 2020 Jun 2;9(6):708. doi: 10.3390/plants9060708 (PMC7356568; doi:10.3390/plants9060708)
Supplement: Supplementary file 1 [file plants-09-00708-s001.zip › plants-791857-supplementary-2/S_5 Analysis of deviance tables.docx]

**SUPPLEMENTARY MATERIALS S5**

**Comparative germination ecology of two endemic *Rhaponticum* species (*Asteraceae*) in different climatic zones of the Ligurian and Maritime Alps (Piedmont, Italy)**

Plants

**Valentina Carasso^1, *^, Marco Mucciarelli^2^, Francesco Dovana^2^, Jonas V Müller^3^**

^1^Centro Regionale Biodiversità Vegetale, Ente di gestione delle Aree Protette delle Alpi Marittime, Via S. Anna, 34, 12013 Chiusa di Pesio, Italy; valentina.carasso@virgilio.it

^2^Università di Torino, Department of Life Sciences and Systems Biology, Viale P.A. Mattioli, 25, 10125 Torino, Italy; marco.mucciarelli@unito.it; francescodovana@libero.it

^3^Royal Botanic Gardens Kew, Millennium Seed Bank, Conservation Science, Wakehurst Place, Ardingly, West Sussex, RH17 6TN, United Kingdom; j.mueller@kew.org

*Correspondence: valentina.carasso@virgilio.it

**Table S6a.** Analysis of the deviance table of the GLM to establish the influence of cold stratification, incubation temperature and species and their interactions on the final germination of Rhaponticum seeds. The models were performed with a binomial error and logit link function for the final germination.

| **Factor** | ***df*** | **Dev.** | **Res. *df*** | **Dev. Res.** | ***P*** |
| --- | --- | --- | --- | --- | --- |
| *NULL* |  |  | 1434 | 1489.9 |  |
| Cold stratification (Str) | 1 | 31.09 | 1433 | 1458.8 | 2.465e-08*** |
| Incubation temperature (T) | 1 | 24.84 | 1432 | 1434.0 | 6.243e-07*** |
| Species (Sp) | 1 | 35.84 | 1431 | 1398.1 | 2.135e-09*** |
| Str x T | 1 | 0.38 | 1430 | 1397.7 | 0.539143 |
| Str x Sp | 1 | 8.25 | 1429 | 1389.5 | 0.004079** |
| T x Sp | 1 | 0.74 | 1428 | 1388.8 | 0.390059 |
| Str x T x Sp | 1 | 1.40 | 1427 | 1387.4 | 0.236613 |
| *df*, degrees of freedom; Dev., deviance; Res. *df*, residual degrees of freedom; Dev. Res., residual deviance; the *P*-values are also shown. Significant values are indicated as *** (*P* <0.001), ** (*P*<0.01) and * (*P*<0.05). | | | | | |

**Table S6b.** Analysis of the deviance table of GLM to establish the influence of incubation temperature and species and their interaction on the mean time to germinate (MTG). The models were performed with a gamma error and inverse link function for the MTG.

| **Factor** | ***df*** | **Dev.** | **Res. *df*** | **Dev. Res.** | ***F*** | ***P*** |
| --- | --- | --- | --- | --- | --- | --- |
| Incubation temperature (T) | 1 | 0.5223 | 4 | 0.3590 | 8.8123 | 0.0972 |
| Species (Sp) | 1 | 0.0092 | 3 | 0.3498 | 0.1546 | 0.7321 |
| Sp x T | 1 | 0.2382 | 2 | 0.1116 | 4.0190 | 0.1829 |
| *df*, degrees of freedom; Dev., deviance; Res. *df*, residual degrees of freedom; Dev. Res., residual deviance; the F-value and *P*-values are also shown. Significant values are indicated as *** (*P* <0.001), ** (*P*<0.01) and * (*P*<0.05). | | | | | | |

**Table S6c.** Analysis of the deviance table of the GLM to establish the effects of the cold stratification treatment, incubation temperature and their interactions on the final germination of *R. scariosum* seeds. The models were performed with a binomial error and logit link function for the final germination.

| **Factor** | ***df*** | **Dev.** | **Res. *df*** | **Dev. Res.** | ***P*** |
| --- | --- | --- | --- | --- | --- |
| *NULL* |  |  | 715 | 607.40 |  |
| Cold stratification (Str) | 1 | 1.8 | 714 | 605.56 | 0.1757 |
| Incubation temperature (T) | 1 | 6.3 | 713 | 599.27 | 0.0121* |
| Str x T | 1 | 1.9 | 712 | 597.41 | 0.1732 |
| *df*, degrees of freedom; Dev., deviance; Res. *df*, residual degrees of freedom; Dev. Res., residual deviance; the *P*-values are also shown. Significant values are indicated as *** (*P* <0.001), ** (*P*<0.01) and * (*P*<0.05). | | | | | |

**Table S6d.** Analysis of the deviance table of the GLM to establish the effects of the cold stratification treatment, incubation temperature and their interactions on the final germination of *R. bicknellii* seeds. The models were performed with a binomial error and logit link function for the final germination.

| **Factor** | ***df*** | **Dev.** | **Res. *df*** | **Dev. Res.** | ***F*** | ***P*** |
| --- | --- | --- | --- | --- | --- | --- |
| Cold stratification (Str) | 1 | 37.4 | 716 | 810.15 | 37.21 | 1.736e-09*** |
| Incubation temperature (T) | 1 | 20.3 | 715 | 789.75 | 20.27 | 7.858e-06*** |
| Str x T | 1 | 0.06 | 714 | 789.69 | 0.06 | 0.7994 |
| The F-value and *P*-values are also shown. Significant values are indicated as *** (*P* <0.001), ** (*P*<0.01) and * (*P*<0.05). | | | | | | |
