# Supplementary material for: Comparative Germination Ecology of Two Endemic Rhaponticum Species (Asteraceae) in Different Climatic Zones of the Ligurian and Maritime Alps (Piedmont, Italy)
Source: Plants (Basel). 2020 Jun 2;9(6):708. doi: 10.3390/plants9060708 (PMC7356568; doi:10.3390/plants9060708)

**SUPPLEMENTARY MATERIALS S6**

**Comparative germination ecology of two endemic *Rhaponticum* species (*Asteraceae*) in different climatic zones of the Ligurian and Maritime Alps (Piedmont, Italy)**

Plants

**Valentina Carasso^1, *^, Marco Mucciarelli^2^, Francesco Dovana^2^, Jonas V Müller^3^**

^1^Centro Regionale Biodiversità Vegetale, Ente di gestione delle Aree Protette delle Alpi Marittime, Via S. Anna, 34, 12013 Chiusa di Pesio, Italy; valentina.carasso@virgilio.it

^2^Università di Torino, Department of Life Sciences and Systems Biology, Viale P.A. Mattioli, 25, 10125 Torino, Italy; marco.mucciarelli@unito.it; francescodovana@libero.it

^3^Royal Botanic Gardens Kew, Millennium Seed Bank, Conservation Science, Wakehurst Place, Ardingly, West Sussex, RH17 6TN, United Kingdom; j.mueller@kew.org

*Correspondence: valentina.carasso@virgilio.it

**Figure S5.** Final germination percentage (mean ± SE) of *R.* *scariosum* (white boxes) and *R. bicknellii* (shaded boxes) for different cold stratification periods with incubation temperatures of (a) 10 °C, (b) 15 °C and (c) 20 °C.


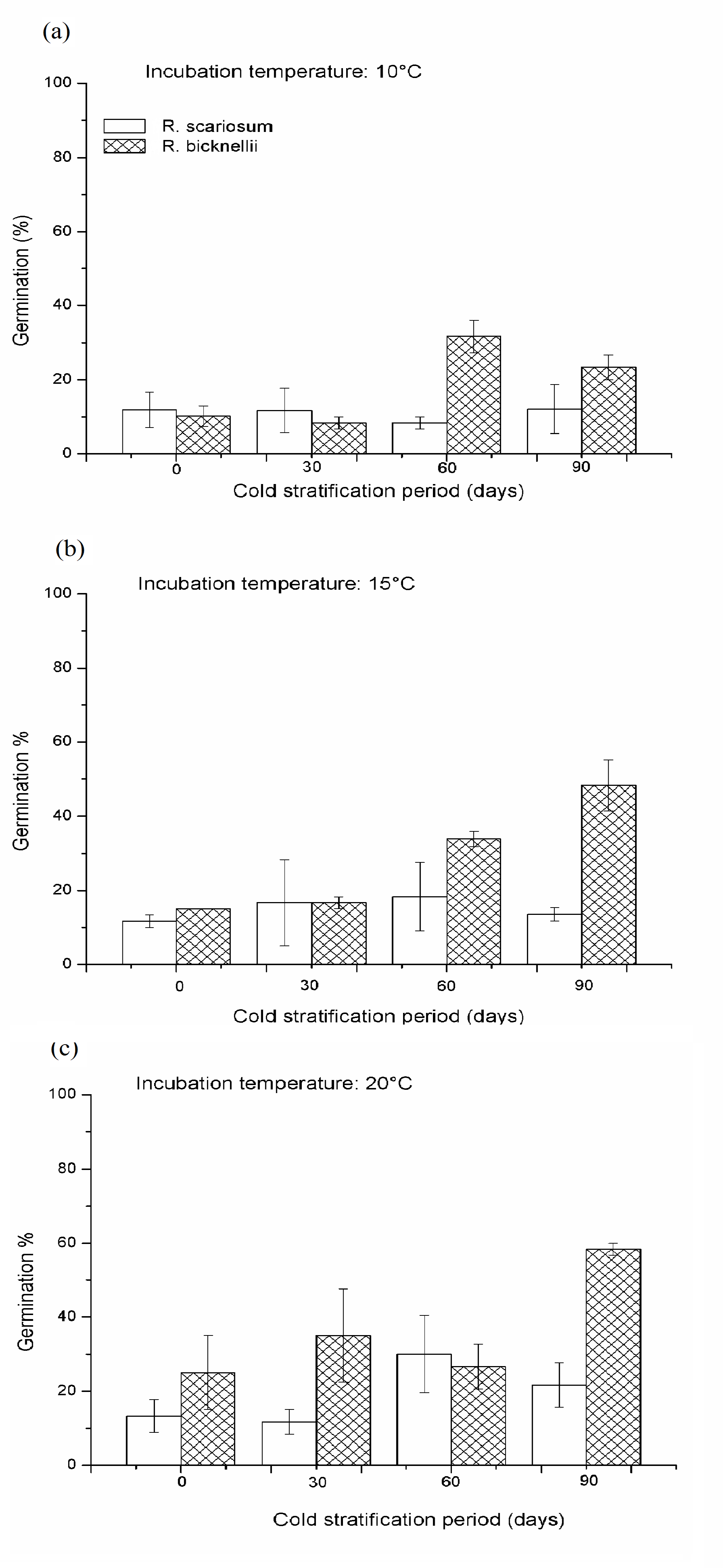

Supplement: Supplementary file 1 [file plants-09-00708-s001.zip › plants-791857-supplementary-2/S_6 Final germination for different cold stratification periods.docx]
