# Supplementary material for: Comparative Germination Ecology of Two Endemic Rhaponticum Species (Asteraceae) in Different Climatic Zones of the Ligurian and Maritime Alps (Piedmont, Italy)
Source: Plants (Basel). 2020 Jun 2;9(6):708. doi: 10.3390/plants9060708 (PMC7356568; doi:10.3390/plants9060708)
Supplement: Supplementary file 1 [file plants-09-00708-s001.zip › plants-791857-supplementary-2/S_8 Weekly soil T and RH during seed dispersal.docx]

**SUPPLEMENTARY MATERIALS S8**

**Comparative germination ecology of two endemic *Rhaponticum* species (*Asteraceae*) in different climatic zones of the Ligurian and Maritime Alps (Piedmont, Italy)**

Plants

**Valentina Carasso^1, *^, Marco Mucciarelli^2^, Francesco Dovana^2^, Jonas V Müller^3^**

^1^Centro Regionale Biodiversità Vegetale, Ente di gestione delle Aree Protette delle Alpi Marittime, Via S. Anna, 34, 12013 Chiusa di Pesio, Italy; valentina.carasso@virgilio.it

^2^Università di Torino, Department of Life Sciences and Systems Biology, Viale P.A. Mattioli, 25, 10125 Torino, Italy; marco.mucciarelli@unito.it; francescodovana@libero.it

^3^Royal Botanic Gardens Kew, Millennium Seed Bank, Conservation Science, Wakehurst Place, Ardingly, West Sussex, RH17 6TN, United Kingdom; j.mueller@kew.org

*Correspondence: valentina.carasso@virgilio.it

| **Table S8**. Mean weekly soil temperatures in °C (± se) and relative humidities (% ± se) during 2015 at the time of seed dispersal and at the beginning of burial experiment for the two study sites (GDC and PDV). | | | | | |
| --- | --- | --- | --- | --- | --- |
| Month | WOY | Soil Temperature (°C) | | Relative Humidity (%) | |
|  |  | GDC | PDV | GDC. | PDV |
| Jul | 29 | 23.06 ± 0.66 | 19.82±2.10 | 51.15±1.75 | 67.11±6.15 |
| Jul | 30 | 20.29 ± 0.57 | 16.42±0.29 | 57.81±3.06 | 56.11±6.92 |
| Jul-Aug | 31 | 18.67 ± 0.88 | **14.31**±0.99 | 64.41±3.79 | **59.61**±9.14 |
| Aug | 32 | 19.14 ± 1.23 | 14.92±1.01 | 64.74±5.53 | 53.42±9.10 |
| Aug | 33 | 14.35 ± 0.89 | 11.22±0.81 | 75.33±3.86 | 78.48±2.69 |
| Aug | 34 | **13.49** ± **0.49** | 11.05±0.47 | **75.68**±3.38 | 51.60±13.10 |
| Aug-Sept | 35 | 16.61 ± 0.42 | 14.26±0.47 | 66.52±3.27 | 75.03±1.36 |
| WOY = week of the year; PDV = Prati del Vallone (CN); GDC = Gola della Chiusetta (CN). Numbers in bold indicate the WOY at which burial experiments were set up. | | | | | |
