# Supplementary material for: Comparative Germination Ecology of Two Endemic Rhaponticum Species (Asteraceae) in Different Climatic Zones of the Ligurian and Maritime Alps (Piedmont, Italy)
Source: Plants (Basel). 2020 Jun 2;9(6):708. doi: 10.3390/plants9060708 (PMC7356568; doi:10.3390/plants9060708)
Supplement: Supplementary file 1 [file plants-09-00708-s001.zip › plants-791857-supplementary-2/S_1 Taxonomic overview of the Rhaponticum species.doc]

**SUPPLEMENTARY MATERIALS S1**

**Comparative germination ecology of two endemic *Rhaponticum* species (*Asteraceae*) in different climatic zones of the Ligurian and Maritime Alps (Piedmont, Italy)**

Plants

**Valentina Carasso^1, *^, Marco Mucciarelli^2^, Francesco Dovana^2^, Jonas V Müller^3^**

^1^Centro Regionale Biodiversità Vegetale, Ente di gestione delle Aree Protette delle Alpi Marittime, Via S. Anna, 34, 12013 Chiusa di Pesio, Italy; valentina.carasso@virgilio.it

^2^Università di Torino, Department of Life Sciences and Systems Biology, Viale P.A. Mattioli, 25, 10125 Torino, Italy; marco.mucciarelli@unito.it; francescodovana@libero.it

^3^Royal Botanic Gardens Kew, Millennium Seed Bank, Conservation Science, Wakehurst Place, Ardingly, West Sussex, RH17 6TN, United Kingdom; j.mueller@kew.org

*Correspondence: valentina.carasso@virgilio.it

| **Table S1.** Taxonomic overview of the *Rhaponticum* species endemic to the western Alps. The taxonomical treatment adopted in this study is based on [27], although other notable classifications are also reported. | | |
| --- | --- | --- |
| Authors | Name | Synonyms |
| Banfi *et al.* 2011 [27] | 1. *Rhaponticum scariosum* Lam., Fl. Franç. (Lamarck) 2:38. 1779. | 1. *Rhaponticum scariosum* Lam. subsp. *lamarckii* (Dittrich) Greuter, Willdenowia 33(1):61. 2003. |
|  | 2. *Rhaponticum bicknellii* (Briq.) Banfi, Galasso & Soldano, Atti Soc. Ital. Sci. Nat. Mus. Civico Storia Nat. Milano 152(2):97. 2011 | 1. *Centaurea rhapontica* L. var. *bicknellii* Briq, Monogr. Centaurées Alpes Marit. 65. 1902. 2. *Rhaponticum heleniifolium* Godr. & Gren. subsp. *bicknellii* (Briq.) Greuter, Willdenowia 33(1):61. 2003. |
|  | 3. *Rhaponticum heleniifolium* Gren. & Godr., Fl. France 2:239. 1850. | -- |
| Pignatti, 1982 [28] | 1. *Rhaponticum scariosum* Lam. subsp. *scariosum* | -- |
|  | 2. *Rhaponticum scariosum* Lam. subsp. *bicknellii* (Gugler) Pignatti, Giorn. Bot. Ital. 111(1-2):57. 1977. | 1. *Centaurea rhapontica* L. var. *bicknellii* Briq, Monogr. Centaurées Alpes Marit. 65. 1902. |
| Tison and de Foucault, 2014 [29] | 1. *Rhaponticum scariosum* Lam. subsp. *scariosum* | 1. *Stemmacantha rhapontica* (L.) Dittrich subsp. *lamarckii* Dittrich, Candollea 45(2):491. 1990. |
|  | 2. *Rhaponticum heleniifolium* Godr. & Gren. subsp. *bicknellii* (Briq.) Greuter, Willdenowia 33(1):61 (2003) | 1. *Leuzea rhapontica L. Holub subsp. bicknellii (Briq.) Holub, Folia Geobot. Phytotax.* 8(4):392. 1973 2. *Stemmacantha heleniifolia (Godr. & Gren.) Dittrich subsp. bicknellii (Briq.) Dittrich, Candollea* 39(1): 47. 1984. |
|  | 3. *Rhaponticum heleniifolium* Godr. & Gren. subsp. *heleniifolium* | -- |
| Global Compositae Checklist, 2017 [30] | 1. *Rhaponticum scariosum* Lam. subsp. *scariosum* | -- |
|  | 2. *Rhaponticum scariosum* subsp. *bicknellii* (Gugler) Pignatti, Giorn. Bot. Ital. 111(1-2):57. 1977. | 1. *Rhaponticum heleniifolium* subsp. *bicknellii* (Briq.) Greuter |
