# Supplementary material for: Comparative Germination Ecology of Two Endemic Rhaponticum Species (Asteraceae) in Different Climatic Zones of the Ligurian and Maritime Alps (Piedmont, Italy)
Source: Plants (Basel). 2020 Jun 2;9(6):708. doi: 10.3390/plants9060708 (PMC7356568; doi:10.3390/plants9060708)
Supplement: Supplementary file 1 [file plants-09-00708-s001.zip › plants-791857-supplementary-2/S_7 Outline of the seed burial experiments in the field.doc]

**SUPPLEMENTARY MATERIALS S7**

**Comparative germination ecology of two endemic *Rhaponticum* species (*Asteraceae*) in different climatic zones of the Ligurian and Maritime Alps (Piedmont, Italy)**

Plants

**Valentina Carasso^1, *^, Marco Mucciarelli^2^, Francesco Dovana^2^, Jonas V Müller^3^**

^1^Centro Regionale Biodiversità Vegetale, Ente di gestione delle Aree Protette delle Alpi Marittime, Via S. Anna, 34, 12013 Chiusa di Pesio, Italy; valentina.carasso@virgilio.it

^2^Università di Torino, Department of Life Sciences and Systems Biology, Viale P.A. Mattioli, 25, 10125 Torino, Italy; marco.mucciarelli@unito.it; francescodovana@libero.it

^3^Royal Botanic Gardens Kew, Millennium Seed Bank, Conservation Science, Wakehurst Place, Ardingly, West Sussex, RH17 6TN, United Kingdom; j.mueller@kew.org

*Correspondence: valentina.carasso@virgilio.it

| **Table S7.** Dates (DD.MM.YY) and duration of the seed burial experiments carried out at Prati del Vallone (PDV) for *R. scariosum* and at Gola della Chiusetta (GDC) for *R. bicknellii*. | | | | |
| --- | --- | --- | --- | --- |
| Species | Dates of seed collection | Dates of field sowing | Dates of seed exhumation | Total duration of seed burial experiment |
| *R. scariosum* | 05.08.2015 | 05.08.2015 | 01.08.2016 | 361 days |
| *R. bicknellii* | 20.08.2015 | 20.08.2015 | 17.08.2016 | 362 days |
